# circ5615 functions as a ceRNA to promote colorectal cancer progression by upregulating TNKS

**DOI:** 10.1038/s41419-020-2514-0

**Published:** 2020-05-11

**Authors:** Zhifei Ma, Chencheng Han, Wenjia Xia, Siwei wang, Xiang Li, Panqi Fang, Rong Yin, Lin Xu, Liu Yang

**Affiliations:** 1 Department of Surgery, The Affiliated Cancer Hospital of Nanjing Medical University & Jiangsu Cancer Hospital & Jiangsu Institute of Cancer Research, Jiangsu Key Laboratory of Molecular and Translational Cancer Research, Nanjing, China; 2 Department of Colorectal Surgery, The Affiliated Cancer Hospital of Nanjing Medical University & Jiangsu Cancer Hospital & Jiangsu Institute of Cancer Research, Nanjing, China

**Keywords:** Genetics research, Colorectal cancer

## Abstract

Circular RNAs (circRNAs), non-coding RNAs generated by precursor mRNA back-splicing of exons, have been reported to fulfill multiple roles in cancer. However, the role of quite a lot circRNAs in colorectal cancer (CRC) remains mostly unknown. Herein, we explored the expression profiles of circRNAs in 5 paired samples of CRC patients by microarray and noted a circRNA, hsa_circ_0005615 (circ5615), was significantly upregulated in CRC tissues. Circ5615 was derived from exon 2 of *NFATC3* and its upregulation was tightly correlated with higher T stage and poor prognosis in CRC patients. Studies in vitro and in vivo demonstrated that knockdown of circ5615 in cancer cells inhibited proliferation and cell cycle acceleration, while overexpression promoted malignant phenotypes. Mechanistically, RNA immunoprecipitation, biotin-coupled probe pull-down and luciferase reporter assays revealed circ5615 effectively bound to miR-149-5p and might play a role like miR-149-5p sponge. Additionally, tankyrase (TNKS), regulator of β-catenin stabilization, was identified as circ5615 downstream and the potential miR-149-5p targets by RNA-seq and bioinformatics analysis. We further verified the upregulation of β-catenin and cyclin D1 induced by circ5615. Our results indicated that circ5615 exerted oncogenic function as competing endogenous RNA (ceRNA) of miR-149-5p to release TNKS and activated Wnt/β-catenin pathway.

## Introduction

Colorectal cancer (CRC) is the third most common cancer with over 1.8 million new cases in 2018 and the second leading cause of cancer-related death worldwide^1^. In addition to genetic risk, lifestyle factors of physical activity, sedentary behavior, and diet show conclusive associations with colorectal cancer incidence^2^. On the therapeutic front, although encouraging evolvement in traditional surgery, radiotherapy, and chemotherapy, a large proportion of patients with advanced CRC are still suffering poor prognosis owing to metastasis and recurrence. Emerging immune checkpoint inhibitors appear promising for cancer treatment, but only ~15% of CRC patients reveal durable response to immunotherapy^3^. Consequently, existing circumstances highlight the necessity to further address underlying molecular mechanisms contributing to the development and progression of CRC to provide more effective treatment options.

Circular RNAs (circRNAs) contain a large class of non-coding RNAs that are produced from precursor mRNA (pre-mRNA) backsplicing, in which a downstream splice-donor site is covalently connected to an upstream splice-acceptor site^4^. With further study of characterization and biology of circRNAs, it has been found that circRNAs generally exhibit cell-type-specific and tissue-specific patterns and are implicated in various diseases such as diabetes mellitus, cardiovascular diseases, and cancer^5^. Due to their circular structures being resistant to most RNA degradation machineries, circular RNAs are deemed to be stable and show bright prospects in clinical application^6^. To date, it has been widely proposed that the circRNAs can act as competing endogenous RNA (ceRNA) for miRNAs through their binding sites and modulate the activity of miRNAs on target genes. CiRS-7, one of the most well-characterized circRNA, with more than 70 conserved binding sites for miR-7, strongly restrains miR-7 activity, bringing about increased expression of miR-7 targets^7^. CircCCDC66 and hsa_circ_101555 also function as miRNA sponge in CRC^8,9^. Additionally, circRNAs interact with RNA-binding proteins acting as protein sponges to regulate protein function^10,11^. A subset of circRNAs undergo cap-independent translation under specific conditions^12,13^. Furthermore, circRNAs might serve as a biomarker for prognosis predication^14^.

Herein, we investigated the expression profiling of circRNA in five CRC and paired adjacent tissues through microarray and found that hsa_circ_0005615 (circ5615) significantly upregulated in CRC tissues. circ5615, clinically related to CRC, promoted the malignant phenotype including cell proliferation in CRC by serving as a miR-149-5p sponge to upregulate TNKS levels. Our findings indicated that circ5615 exerted oncogenic potential and could be a candidate in diagnosis and treatment of CRC.

## Results

### Expression profiles of circRNAs in colorectal cancer

**Fig. 1:**
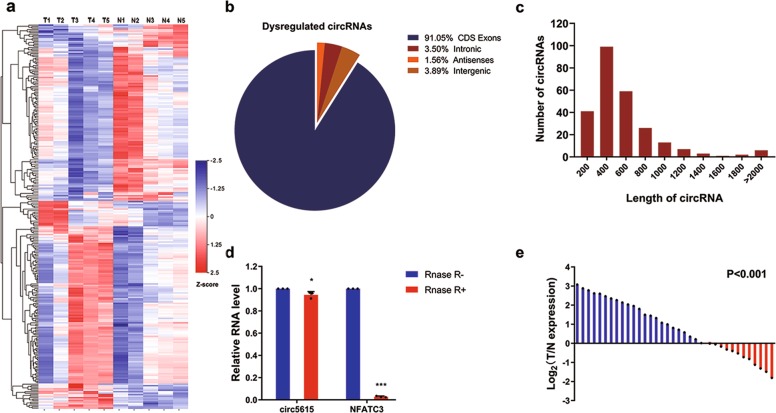
Microarray revealed that circ5615 was upregulated in CRC. **a** Heat map of differentially expressed circRNAs between 5 CRC tissues and adjacent nontumor tissues. Gene expression in *z* score-transformed value was shown. **b** Pie chart showing dysregulated circRNAs derived from different genomic regions. **c** Length distribution of the dysregulated circRNAs. **d** The PCR analysis validated that circ5615 resisted to RNase R, while corresponding linear NFATC3 mRNA could be digested by RNase R. **e** Expression of circ5615 in 35 paired CRC samples were detected by RT-PCR. *ACTB* was used as a loading control. T tumor tissue, N nontumorous tissue. Data are shown as mean ± SD. *p < 0.05; ***p < 0.001, paired *t*-test.

To analyze the expression profiles of circRNA in colorectal cancer (CRC), we performed microarrays in five paired samples of CRC and adjacent nontumor tissues (GSE142837), which contained 3314 circRNA probes. Subsequently, a total of 257 dysregulated circRNAs were identified in colorectal cancer tissues, of which 139 circRNAs were upregulated and 118 circRNAs were downregulated compared to the adjacent nontumor tissues (Fig. 1a and Supplementary Fig. 1a). Among these 257 circRNAs, 91.05% were derived from exons (Fig. 1b), and the majority were <1200 nucleotides (nt) (Fig. 1c). Focus on the potential oncogene in CRC, we next analyzed the most up-regulated circRNAs expression by reverse transcription polymerase chain reaction (RT-PCR) after RNase R treatment to check the resistance of circRNAs to RNase R digestion. Results showed that hsa_circ_0005615, hsa_circ_0000467, and hsa_circ_0045932 were stable while their linear isoform could be easily digested by RNase R (Fig. 1d and Supplementary Fig. 1b). Hsa_circ_0048232 could not be detected due to their low abundance in CRC cells. For the others, we tried different primers but all of these primers caused nonspecific amplification, mainly because of alternative splicing of circRNAs. Further assessment of the three circRNAs in 35 CRC tissues by RT-PCR, we found them markedly up-regulated in CRC tissues compared to adjacent nontumor tissues (Fig. 1e and Supplementary Fig. 1c, d). These data matched well with the microarray data, indicating the high reliability of the microarray results. Considering that hsa_circ_0005615 (circ5615) was significantly upregulated (2.8-fold change), whose expression was much higher than hsa_circ_0000467 and hsa_circ_0045932 (more than nine times, *p* < 0.01), we chose circ5615 for further investigation.

### Characterization of circ5615 in colorectal cancer

**Fig. 2:**
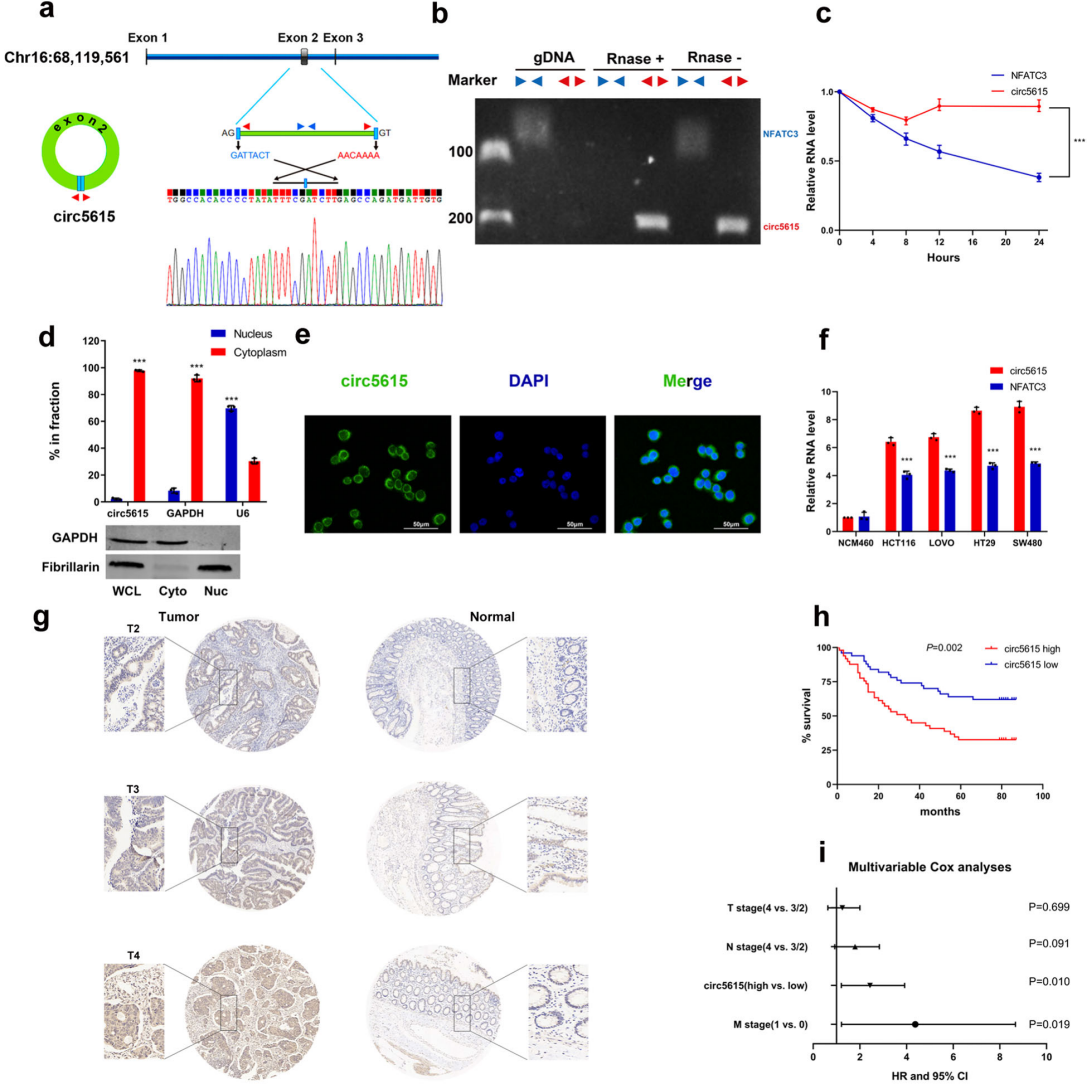
Characterization of circ5615 in CRC cells. **a** circ5615 was back- spliced by exon 2 of NFATC3, confirmed by sanger sequencing. **b** Divergent and convergent primers were designed and the gene expression with or without RNase R treatment was detected by gel electrophoresis. **c** RNA stability of circ5615 and NFATC3. Hours: time after ActD treatment. **d** RT-PCR analysis and western blot of subcellular fractionation in the SW480 cells. Fibrillarin and GAPDH served as a specific nuclear and cytoplasmic marker to whole-cell lysates (WCL), cytoplasm (Cyto), and nucleus (Nuc). **e** Confocal microscopy images of circ5615 (green) in SW480 cells. Nuclei was stained with DAPI (blue). **f** circ5615 and NFATC3 mRNA expression in CRC cell lines and human normal colorectal cell line. **g** The expression of circ5615 analyzed by CISH on CRC tissues was correlated with T stage. **h** Kaplan–Meier analysis of the correlation between circ5615 levels and overall survival. High expression of circ5615 led to a significantly shorter overall survival. **i** Multivariable analysis showed high circ5615 level was an independent prognostic factor in CRC. Data are shown as mean ± SD. *p < 0.05; **p < 0.01; ***p < 0.001, paired *t*-test.

Circ5615 was circularized by exon 2 of the *NFATC3* gene with a length of 1135 nt according to circBase (http://www.circbase.org). We designed divergent primers amplifying the back-spliced junction of circ5615 and Sanger sequencing was used to confirm the circ5615 junction (Fig. 2a). After RNase R treatment, the divergent primers could detect circ5615, which is resistant to digestion by RNase R, while the divergent primers could not amplify any products in genomic DNA. In contrast, convergent primers specifically for *NFATC3* mRNA amplified the linear mRNA, which disappeared after RNase R digestion (Fig. 2b). Further analysis for stability of circ5615 with SW480 cells treated with Actinomycin D, an inhibitor of transcription, showed that the half-life of circ5615 transcript exceeded 24 h (Fig. 2c). Repetitive elements residing in introns flanking circularized exons, such as Alu elements in primates, have been reported to be responsible for most circRNA formation^15^. The analysis of the flanking introns of *NFATC3* exon 2 revealed highly complementary Alu repeats with 37 short interspersed elements in the intron upstream of *NFATC3* exon 2 and 6 short interspersed elements downstream (Supplementary Fig. 1e). The inverted repeated Alu elements (IRAlus) are highly reverse complementary (typically 84% identity over 281 nt; Supplementary Fig. 1e), probably contributing to the elevated expression of circ5615. Additionally, the expression of circ5615 was positively correlated with *NFATC3* (*r* = 0.7328, *p* < 0.001, Supplementary Fig. 1f), implying that higher expression of circ5615 was relevant with higher expression of *NFATC3*.

To evaluate the subcellular localization of circ5615 in CRC cells, we used the RT-PCR analysis to determine the nuclear and cytoplasmic circ5615 expressions respectively. Our results revealed that circ5615 preferentially located in the cytoplasm of SW480 cells (Fig. 2d), which was also identified by the fluorescence in situ hybridization (FISH) assay for circ5615 (Fig. 2e). Additionally, we observed that circ5615 expression increased in CRC cell lines compared with human normal colorectal cell line (Fig. 2f), which was consistent with the results in CRC tissues. Based on this result, SW480 and HCT 116 cells were selected for the following circ5615 assays. Moreover, the levels of circ5615 was 1.72-fold change in CRC cell lines and 2.64-fold change in CRC tissues compared to *NFATC3* (Supplementary Fig. 1g).

### Circ5615 expression correlated with poor clinical outcome

We then explored the clinicopathologic significance of circ5615 using tissue microarray (TMA) constructed by 99 pairs of CRC tissues and adjacent nontumor tissues. Specific digoxigenin-labeled probe was designed to detect circ5615 expression by chromogenic in situ hybridization (CISH). High expression of circ5615 in CRC was also validated by immunoreactive scores in TMA, which was significantly correlated with higher T stage in CRC patients (Fig. 2g and Table 1). Kaplan–Meier survival curves revealed that CRC patients with high circ5615 levels had a shorter overall survival (HR = 2.331, *P* = 0.002; Fig. 2h). Additionally, multivariable analysis indicated that circ5615 level was an independent prognosis factor for CRC patients (HR = 2.176, *P* = 0.010; Fig. 2i). Thus, the results demonstrated that circ5615, a stable circRNA, whose upregulation was common in CRC, led to poor prognosis.

**Table 1:** The relationship between circ5165 and clinical characteristics in 99 CRC patients.

Variable	circ5615		*p*-value
	Low (*n* = 50)	High (*n* = 49)	
Sex			
Male	25	34	0.0494*
Female	25	15	
Age			
>60	40	36	0.4417
≤60	10	13	
T stage			
II–III	39	28	0.0265*
IV	11	21	
N stage			
0	28	24	0.4843
I–II	22	25	
M stage			
0	48	46	0.6297
I	2	3	

### Circ5615 promoted the malignant phenotype of colorectal cancer cell lines in vitro

**Fig. 3:**
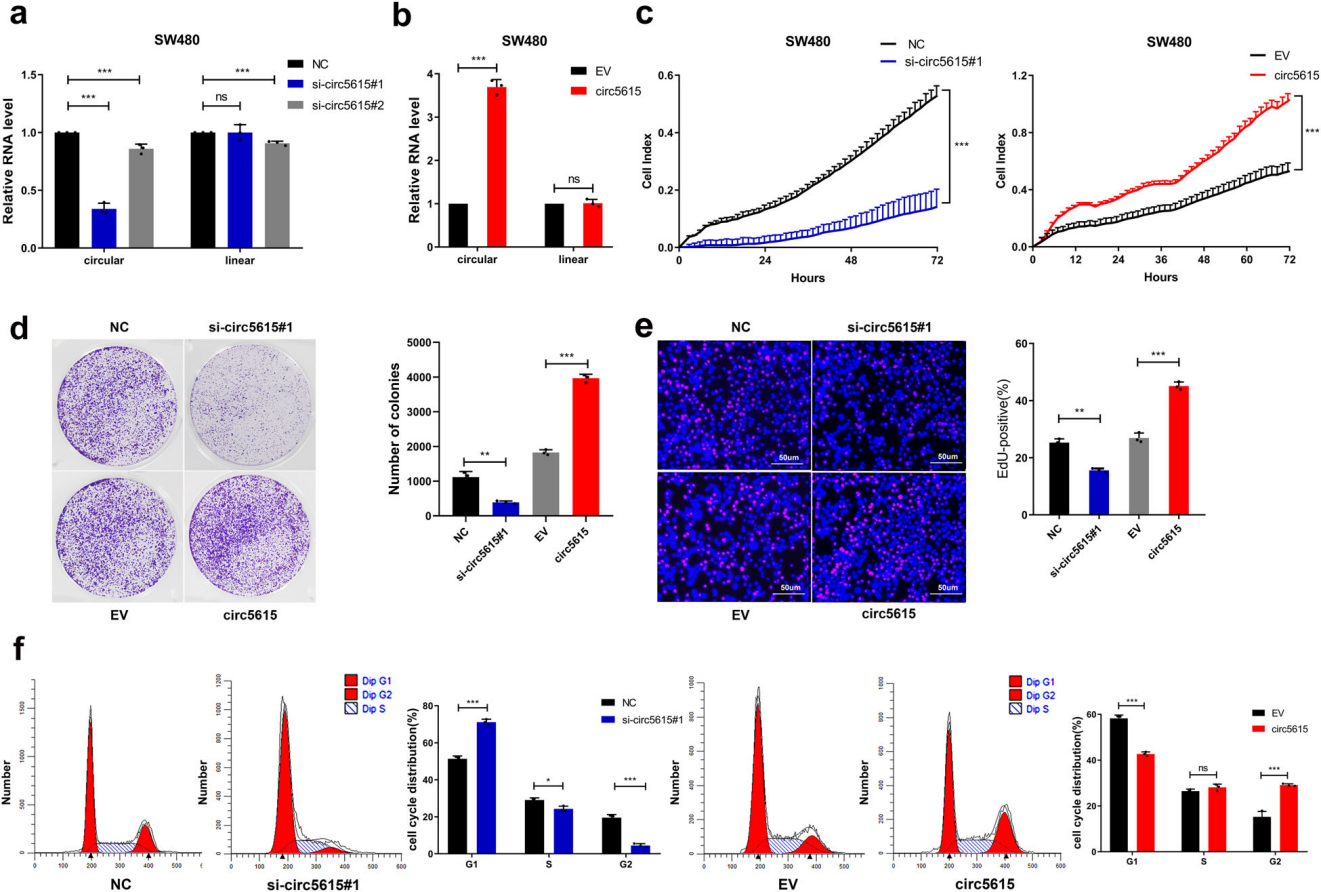
Expression of circ5615 promoted cell proliferation in CRC cells. **a** The interfering efficiencies of siRNA in SW480 cells were determined by RT-PCR. **b** Expression levels of circ5615 and NFATC3 in SW480 cells after transduction with circ5615 vector. **c**–**e** Circ5615 facilitated the proliferation of CRC cells shown by the RTCA (**c**), colony formation (**d**), and EdU (**e**) assays with silencing or overexpressing circ5615. Cell numbers were determined using the ImageJ program. **f** Cell cycle analysis of SW480 cells with silencing or overexpressing circ5615. Data are shown as mean ± SD (*n* = 3) or typical photographs of one representative experiment. Similar results were obtained in three independent experiments. *p < 0.05; **p < 0.01; ***p < 0.001; ns nonsignificant, paired *t*-test.

To investigate the role of circ5615 in CRC progression, we designed two short interfering RNAs (si-circ5615#1 and si-circ5615#2), which specifically target the back-splicing region of circ5615. Then a strict control RNA (NC) with half-sequence replacements of siRNA was designed to exclude potential off-target effects^13^. For overexpression of circ5615, exon 2 of *NFATC3* was cloned into the expression vectors, together with upstream and downstream flanking intronic sequences to promote the formation of circ5615 as in a previous study^16^. Compared with the control siRNA, si-circ5615#1 rather than si-circ5615#2 significantly downregulated the expression of circ5615 but not *NFATC3* in SW480 and HCT 116 cells so we chose si-circ5615#1 for following assays (Fig. 3a and Supplementary Fig. 2a). The overexpression vector significantly increased the expression of circ5615 as opposed to the empty vector while *NFATC3* mRNA expression had no obvious change in both CRC cells (Fig. 3b and Supplementary Fig. 2b). The results demonstrated that circ5615 could not affect the expression of *NFATC3*. By real-time cell analyzer system (RTCA), colony formation, and 5-ethynyl-2′-deoxyuridine (EdU) proliferation assays, we determined that knockdown of circ5615 remarkably impaired the proliferation ability of SW480 and HCT 116 cells, whereas ectopic expression of circ5615 promoted cell viability (Fig. 3c–e and Supplementary Fig. 2c). We next evaluated whether circ5615 affects apoptosis or cell-cycle progression of SW480 cells. Cell cycle analysis illustrated that CRC cells were arrested at G1 phase after knockdown of circ5615. Conversely, stable overexpression of circ5615 caused the progression of cells from the G1/S to G2/M phase (Fig. 3f). The results of Annexin-V-PE staining showed circ5615 had no significant effect on CRC cells apoptosis, implying circ5615 promoted cell proliferation in a cell-cycle-dependent manner (Supplementary Fig. 2d). Additionally, the Transwell and Matrigel assays revealed that circ5615 induced the invasion of SW480 cells (Supplementary Fig. 2e, f). Both gain-of-function and loss-of-function experiments in vitro suggested that circ5615 might play an oncogenic role in CRC.

### Circ5615 served as a sponge for miR-149-5p

**Fig. 4:**
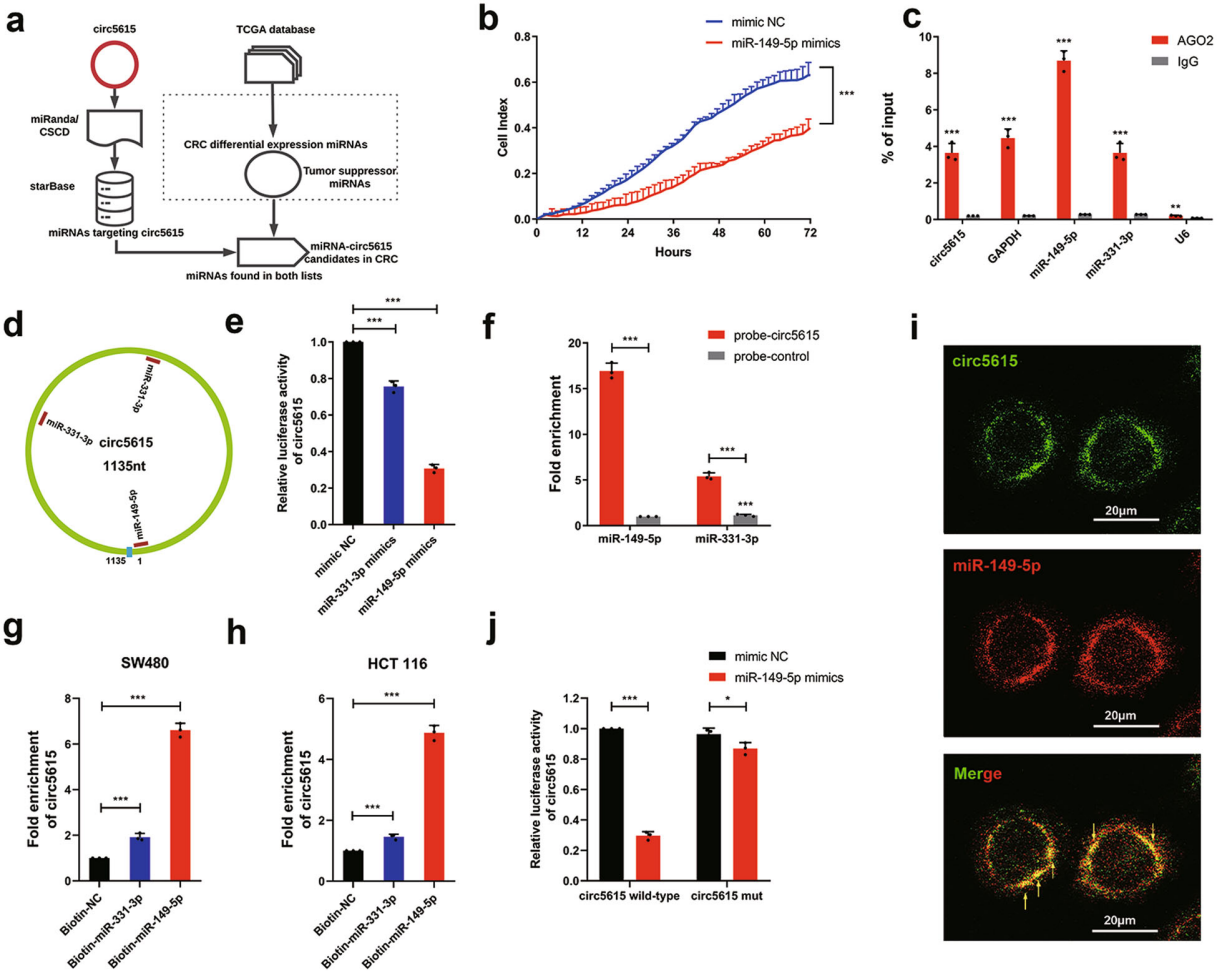
Identification of miR-149-5p binding to circ5615. **a** Schematic flowchart illustrated the pipelines for miRNA analyses. **b** RTCA proliferation analysis for SW480 cells transfected with miR-149-5p mimics. **c** The Ago2 RIP showed that Ago2 served as a platform for circ5615, miR-149-5p and miR-331-3p. **d** Schematic illustration showed the potential binding sites of miR-149-5p and miR-331-3p on circ5615. **e** Luciferase activity of circ5615 transfected with miR-149-5p or miR-331-3p mimics. Firefly luciferase activity was normalized by Renila luciferase activity. **f** Biotin-coupled CircRNA pull-down assays confirmed that miR-149-5p was effectively enriched by circ5615 in SW480 cells. **g**, **h** circ5615 was enriched with 3′-end biotinylated miR-149-5p in SW480 and HCT 116 cells. **i** The FISH assays showed that circ5615 and miR-149-5p were relatively co-localized in the cytoplasm of SW480 cells. **j** Luciferase reporter activity of circ5615 co-transfected with miR-149-5p mimics or mimics NC. Data are shown as mean ± SD (*n* = 3). Similar results were obtained in three independent experiments. *p < 0.05; **p < 0.01; ***p < 0.001, paired *t*-test.

Because the internal ribosome entry sites (IRESs) parameter index <1.6 and circ5615 had a short open reading frame, we predicted the relatively low protein coding potential of circ5615^17,18^. Recent studies have shown that circRNAs can function as miRNA sponges to modulate downstream targets expression^7,19–21^. Given that circ5615, the circRNA highly expressed in CRC, mainly located in the cytoplasm, we next explored miRNAs targeting circ5615 via the competitive endogenous (ceRNA) dependent mechanism. The analysis pipeline was detailed in Fig. 4a. Briefly, miRanda and CSCD target prediction tools revealed that 185 miRNAs were found to have the potentially binding sites to circ5615^22,23^. AGO CLIP-seq data from starBase database was used for identification of miRNA-circ5615 interactions^24^. Moreover, we filtered tumor-suppressor miRNAs with significantly low expression in CRC by analysis of The Cancer Genome Atlas (TCGA) database (Supplementary Fig. 3a). Consequently, three potentially functional candidate miRNAs (miR-149-5p, miR-331-3p and miR-3944-3p) were found in both lists. By RTCA proliferation assays, we observed miR-149-5p, miR-331-3p suppressed the proliferation ability of SW480 cells, but not miR-3944-3p (Fig. 4b and Supplementary Fig. 3b, c). We next performed AGO2 RNA immunoprecipitation assays (RIP) in SW480 cells to explore whether AGO2 could bind with both circ5615 and miRNAs. Indeed, endogenous circ5615, miR-149-5p and miR-331-3p were efficiently enriched by anti-AGO2 compared to anti-IgG (Fig. 4c, d), which verified the validity of our analysis.

To further explore the binding capability of miR-149-5p and miR-331-3p to circ5615, luciferase reporters were constructed and showed that the luciferase activities of circ5615 was significantly reduced by nearly 70% transfected with miR-149-5p mimics; while only 30% transfected with miR-331-3p mimics (Fig. 4e). Similar results were observed in circRNA pull-down assays in SW480 cells (Fig. 4f), suggesting miR-149-5p had stronger ability to sponge circ5615. We then performed miRNA pull-down assays using biotin-coupled miRNA mimics and determined that circ5615 was significantly enriched by miR-149-5p rather than miR-331-3p (Fig. 4g, h). The FISH results demonstrated that circ5615 and miR-149-5p were relatively co-localized in the cytoplasm of CRC cells (Fig. 4i). More importantly, the reporter plasmid in which the predicted miR-149-5p binding sites were mutated revealed that transfection with miR-149-5p mimics significantly inhibited the activity of circ5615 wild-type reporter, but not mutated luciferase reporter (Fig. 4j and Supplementary Fig. 3d). Therefore, these findings implied circ5615 served as a sponge for miR-149-5p which suppressed the tumorigenesis of CRC cells.

### MiR-149-5p decreased TNKS expression

**Fig. 5:**
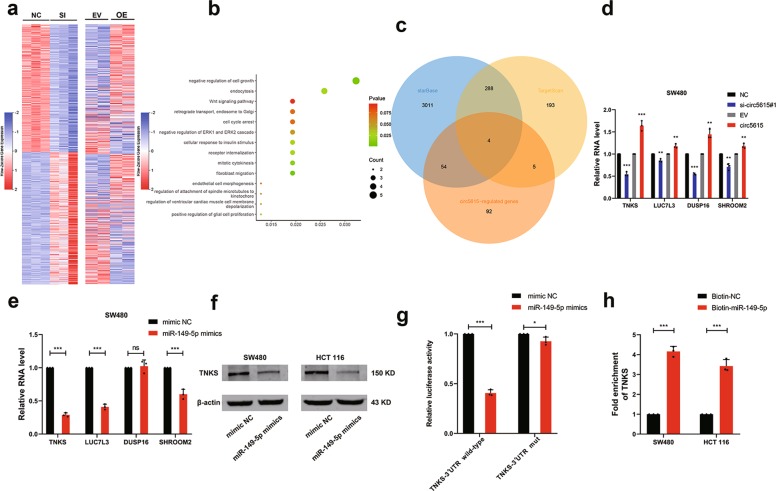
TNKS was identified as a direct target of miR-149-5p. **a** Heat map analyses showed top 500 activated and suppressed genes by RNA-seq (left) and corresponding gene expression by microarray (right). SI: circ5615 knockdown. OE: circ5615 overexpression. **b** The GO biological processes analysis of the circ5615-regulated genes (fold changes >1.41 in RNA-seq samples and <0.80 in microarray samples; fold changes <0.70 in RNA-seq samples and >1.20 in microarray samples, *p* < 0.05). **c** Four target genes were showed with the overlap between circ5615-regulated genes and predicted miR-149-5p targets. **d** The expression of genes after circ5615 knockdown or overexpression in SW480 cells. **e** RT-PCR analysis revealed TNKS, LUC7L3 and SHROOM2 expression were significantly decreased by miR-149-5p in SW480 cells. **f** The protein levels of TNKS in the CRC cells transfected with miR-149-5p mimics. **g** Luciferase reporter activity of TNKS-3′UTR wild-type-reporter or mutant- reporter in HEK-293T cells transfected with miR-149-5p mimics or mimic NC. **h** Biotin-coupled miRNA pull-down assays determined that TNKS was effectively enriched by miR-149-5p. Data are shown as mean ± SD (*n* = 3). Similar results were obtained in three independent experiments or typical photographs of one representative experiment. *p < 0.05; **p < 0.01; ***p < 0.001; ns nonsignificant, paired *t*-test.

To better understand the molecule mechanism of circ5615 and regulatory role of miR-149-5p in CRC, we performed RNA-seq in SW480 cells after silencing circ5615, which was verified by mRNA microarray after circ5615 overexpression. The top 500 genes significantly downregulated in knockdown samples were upregulated in overexpression samples and vice versa (Fig. 5a), suggesting that phenotypes observed above were not caused by off-target effects. Gene Ontology enrichment analysis of the remarkedly dysregulated genes showed the top biological processes were cell growth related, including Wnt signaling pathway^25^ (Fig. 5b). By TargetScan and starBase miRNA prediction programs^24,26^, we identified the target sequences of miR-149-5p in four genes 3′-UTR (*TNKS*, *LUC7L3*, *DUSP16*, and *SHROOM2*), whose changes in RNA levels were verified to be consist with circ5615 (Fig. 5c, d and Supplementary Fig. 4a). Further RT-PCR analysis revealed *TNKS, LUC7L3*, and *SHROOM2* expression significantly decreased when transfected with miR-149-5p mimics (Fig. 5e and Supplementary Fig. 4b). Considering that *TNKS* expression changed the most after circ5615 knockdown or overexpression, we chose *TNKS* for further verification, observing the protein levels of TNKS were decreased in CRC cells transfected with miR-149-5p mimics (Fig. 5f). Tankyrase (TNKS) has been reported to modulate a diverse range of processes involving regulation of the Wnt signaling pathway through β-catenin destruction and control of the mitotic checkpoint^27^. To further explore whether the 3′-UTR of *TNKS* was a functional target of miR-149-5p, we cloned the wild-type and mutant (predicted miR-149-5p binding sites were mutated) 3′-UTR of *TNKS* mRNA and performed dual luciferase reporter assays. Compared with the control RNA group, miR-149-5p mimics efficiently reduced luciferase activity of wild-type group but not mutant one (Fig. 5g and Supplementary Fig. 4c). Furthermore, miRNA pull-down assay showed a nearly four-fold enrichment of *TNKS* in the miR-149-5p group compared with the control one (Fig. 5h). These results suggested that miR-149-5p could bind to the 3′-UTR of *TNKS* and directly downregulate TNKS expression.

### Wnt/β-catenin pathway was activated by circ5615 through TNKS

**Fig. 6:**
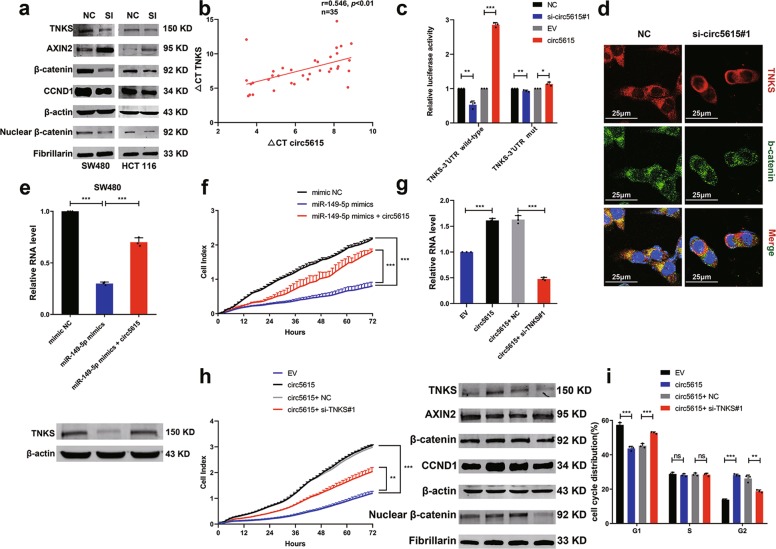
circ5615 promoted cell proliferation by relieving the repression of miR-149-5p for TNKS expression. **a** The protein levels of TNKS, AXIN2, β-catenin, and CCND1 in the CRC cells with knockdown of circ5615. SI: circ5615 knockdown. Fibrillarin served as a specific nuclear marker. **b** Positive correlation between the levels of circ5615 and TNKS of the 35 CRC tumorous tissues. ΔCt values were normalized according to *ACTB*. **c** Luciferase reporter activity of TNKS-3′UTR wild-type-reporter or mutant-reporter in HEK-293T cells with the knockdown or overexpression of circ5615. **d** Cellular expression of TNKS and β-catenin in HCT 116 cells after circ5615 knockdown. Nuclei was stained with DAPI (blue). **e** The mRNA (upper panel) and protein (lower panel) levels of TNKS expression in SW480 cells transfected with miR-149-5p mimics alone or co-transfected with circ5615. **f** RTCA cell proliferation analysis in SW480 cells transfected with miR-149-5p mimics alone or co-transfected with circ5615. **g** Upper panel: the TNKS mRNA expression in SW480 cells with circ5615 overexpression and TNKS knockdown. Lower panel: the protein levels of TNKS, AXIN2, β-catenin, and CCND1 in SW480 cells with circ5615 overexpression and TNKS knockdown. Fibrillarin served as a specific nuclear marker. **h** RTCA cell proliferation assays in SW480 cells with circ5615 overexpression after TNKS knockdown. **i** Cell cycle analysis of SW480 cells with circ5615 overexpression after TNKS knockdown. Data are shown as mean ± SD (*n* = 3) or typical photographs of one representative experiment. *p < 0.05; **p < 0.01; ***p < 0.001; ns nonsignificant, paired *t*-test.

Since our data showed that circ5615 served as a sponge for miR-149-5p and miR-149-5p bind to the 3′-UTR of *TNKS*, we further verified whether circ5615 induced *TNKS* expression, which had been validated in RNA levels (Fig. 5d and Supplementary Fig. 4a). Firstly, the protein levels of TNKS decreased with circ5615 knockdown, whereas increased when circ5615 was overexpressed (Fig. 6a and Supplementary Fig. 4d). Second, circ5615 expression was positively related with mRNA levels of *TNKS* in the expression cohort of 35 CRC patients (*r* = 0.546, *p* < 0.01; Fig. 6b). Additionally, we noted that the changes of circ5615 expression had little effect on the luciferase activity of *TNKS*-3′UTR mutant-reporter (Fig. 6c).

Previous work has demonstrated TNKS could stabilize β-catenin protein by promoting AXIN2 degradation and implicated in multiple functions including cellular proliferation in CRC^28,29^. GO analysis on genes regulated by circ5615 also implied that circ5615 participated in Wnt signaling pathway (Fig. 5b). Consequently, we performed Western blot analysis and observed circ5615 induced decreased AXIN2 and increased β-catenin, including the raised nuclear β-catenin levels. β-catenin translocating to the nucleus has been reported to promote the transcription of a series of target genes including cyclin D1 (*CCND1*)^30^, which was stimulated by circ5615 (Fig. 6a and Supplementary Fig. 4d), partly explaining the influence of circ5615 on cell cycle. Similar results were observed by the Immunofluorescence assay (Fig. 6d).

### Circ5615 promoted cell proliferation via the circ5615-miR-149-5p-TNKS axis

Then we designed rescue experiments using miR-149-5p mimics in SW480 cells, which showed that miR-149-5p mimics alone significantly decreased *TNKS* expression, but it was rescued by circ5615 overexpression (Fig. 6e and Supplementary Fig. 4f). RTCA proliferation assays revealed circ5615 overexpression could partially rescue miR-149-5p mimics-mediated suppression for proliferation (Fig. 6f and Supplementary Fig. 4g). As shown in Fig. 6g, circ5615 could promote β-catenin nuclear entry activating CCND1 with AXIN2 degradation and *TNKS* siRNA significantly attenuated the effects of circ5615 on AXIN2, β-catenin, and CCND1. Similarly, knockdown of TNKS could diminish the effect of circ5615 overexpression on CRC cells proliferation and cell cycle (Fig. 6h, i). Taken together, we demonstrated that circ5615-regulated CRC cell proliferation via the circ5615-miR-149-5p-TNKS axis.

### circ5615 promoted colorectal cancer tumorigenesis in vivo and served as a potential therapeutic target

**Fig. 7:**
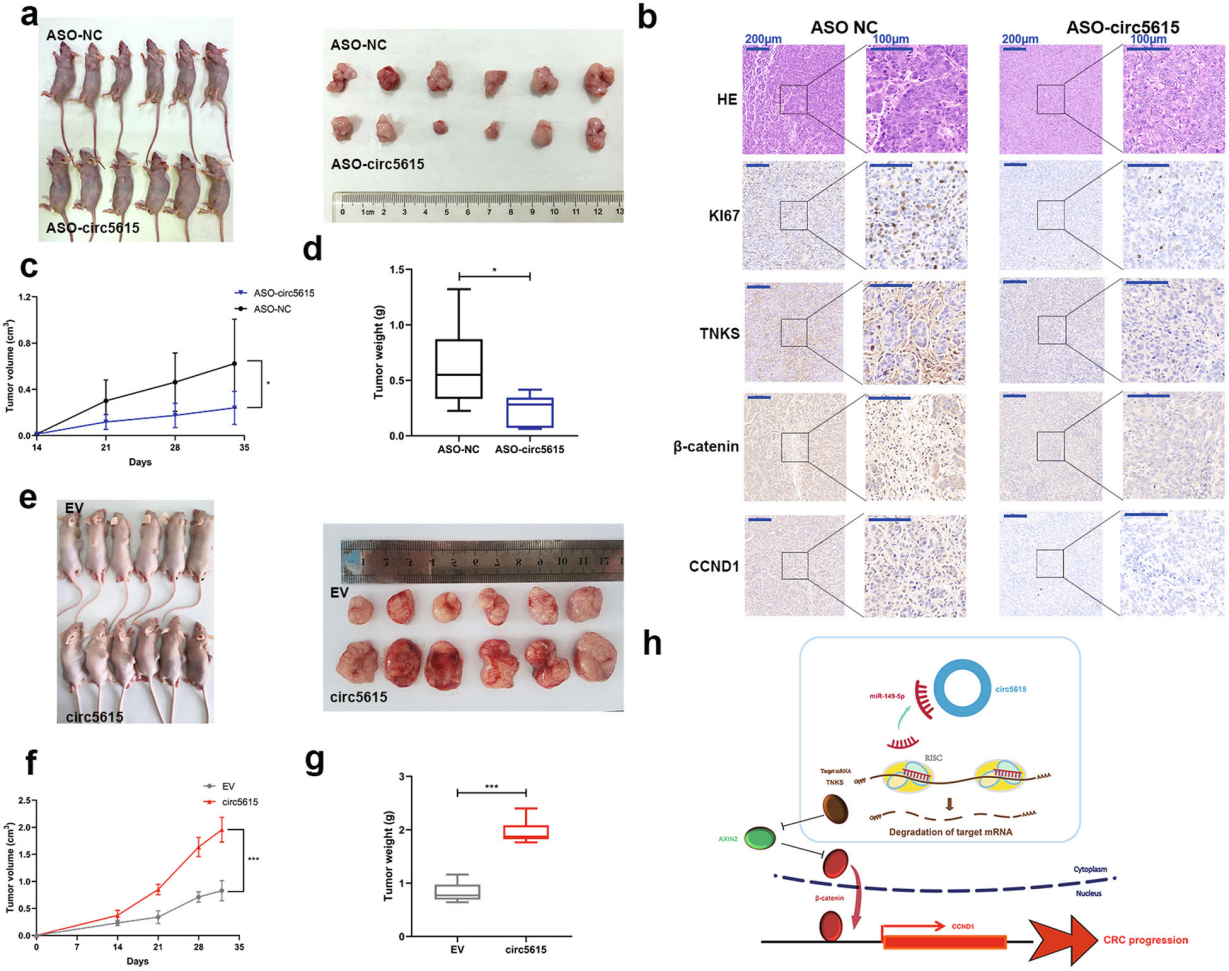
circ5615 promoted tumor growth in vivo. **a** Xenograft tumor models revealed that tumors grown with circ5615 knockdown were smaller than the control group. **b** Serial sections and IHC staining of TNKS, β-catenin, CCND1, and Ki67. **c**, **d** The volume (c) and weight (d) of subcutaneous xenograft tumors from nude mice. **e** Xenograft tumor models showed that circ5615 significantly promoted tumor growth. **f**, **g** The volume (f) and weight (g) of subcutaneous xenograft tumors after overexpression of circ5615. **h** Hypothesis diagram showed function and mechanism of circ5615 in CRC progress. Data are shown as mean ± SD (*n* = 6). In **c**, **d**, **f**, **g**, center line: median of data; Bounds of box: the second quartile to the third quartile; Whisker: minimum value to maximum value. *p < 0.05; ***p < 0.001, paired *t*-test.

To explore the biological function of circ5615 in vivo, we injected circ5615 antisense oligonucleotide (ASO) into established xenograft tumor model in nude mice and noted the circ5615 ASO suppressed tumor growth (Fig. 7a, c, d). Serial sections and immunohistochemical (IHC) staining showed that tumor tissues injected with circ5615 ASO had fewer TNKS, β-catenin, and CCND1-positive cells (Fig. 7b). Correspondingly, tumors derived from cells transfected with circ5615 expression vectors grew more rapidly (Fig. 7e–g). ASO provided specific efficiency in regulating target gene expression and had the potential for clinical treatment^31^. Therefore, the results in vivo suggested that circ5615 could act as a promising therapeutic target of colorectal cancer.

## Discussion

Recent high-throughput expression profiling studies function as an important resource to explore the expression patterns of circRNAs in cancer. Various circRNAs have been found participating in the development and progress of cancer^32–34^. Focus on oncogenes in colorectal cancer, we performed circRNA expression microarray and noted circ5615 as a markedly upregulated circRNA in CRC after RNase R digestion and RT-PCR analysis. Function assays in vitro revealed that circ5615 promoted the proliferation and migration of colorectal cancer cell lines. Circ5615 served as a sponge for miR-149-5p and inhibited the miRNA- mediated suppression on the target gene TNKS. Increased TNKS level drove CRC cell proliferation via Wnt/β-catenin pathway by stimulating AXIN2 degradation and therefore stabilize β-catenin, implying that circ5615 promotes CRC cell growth via a ceRNA-dependent mechanism.

Most circRNAs are generated by precursor mRNA via exon circularization. We characterized one of the abundant circRNAs derived from exon 2 of the *NFATC3* gene (termed circ5615), whose flanking introns consisted of many complementary Alu repeats. The majority of circRNAs, except for intron-containing circRNAs, are localized to cytoplasm^35^. Consisting of a single exon, circ5615 was observed principally exported to the cytoplasm. The stable nature of circ5615 (Fig.2b, c) partly accounted for the elevated expression compared to *NFATC3* both in CRC cell lines and tissues.

Because of the unique properties and diverse cellular functions, circRNAs have great potential as diagnostic and prognostic biomarkers^36^. We then evaluated the clinical relevance of circ5615. Both RT-PCR and CISH analyses revealed circ5615 expression rose in CRC tissues. There was a significantly positive correlation between circ5615 expression and T stage in CRC patients. We also found that male patients had elevated circ5615 expression, probably because of the higher mortality rates in men than in women^37^. Correspondingly, CRC patients with poor prognosis showed increased levels of circ5615, which was further identified an independent prognosis factor for CRC patients. The evidence suggested that circ5615 was a potential biomarker for colorectal cancer. Loss of function and gain-of function assays in vitro revealed circ5615 promoted proliferation in a cell-cycle-dependent manner and induced migration in CRC cell lines, which was concordant with analyses above. As *NFATC3* has been reported to promote the development of intestinal tumors through NFAT-dependent transcriptional regulation^38^, circ5615 along with its host gene could facilitate the CRC progression in cytoplasm and nucleus respectively.

It has been proposed that the competing endogenous RNAs (ceRNAs), including circRNAs, can serve as miRNAs sponges and regulate the activity of miRNAs on target genes^39,40^. We explored the possibility of circ5615 as a ceRNA because of the abundant expression (0.03 of *ACTB* expression and 2.64-fold change in CRC tissues). After multiple bioinformatic analyses and proliferation assays (Fig. 4a, b), miR-149-5p and miR-331-3p were noted as tumor-suppressor miRNAs in CRC with potentially circ5615 binding sites. Subsequently, AGO2 RIP and circRNA/miRNA pull-down assays showed the evidence of the physical interaction between circ5615 and miR-149-5p/miR-331-3p, but miR-149-5p had stronger binding strength than miR-331-3p, corresponding with the results of luciferase reporter assays. In consistent with the results above, miR-149-5p has been reported to function as tumor suppressor in multiple tumors.^41–43^. Our study suggested circ5615 directly bound to miR-149-5p and might serve as a ceRNA.

TNKS, a member of poly (ADP-ribose) polymerase (PARP) family enzymes, implicates in diverse functions including modulation of the Wnt signaling, regulation of telomere length, mediation of mitotic progression and control of insulin-stimulated glucose uptake^27^. High-throughput analysis of potential circ5615-regulated mRNA and further GO enrichment analysis revealed circ5615 was involved in cell growth, mitotic cytokinesis, and cellular response to insulin stimulus (Fig. 5b), indicating the consistency with the function of TNKS. Meanwhile, miRNA prediction algorithm and gene expression validation implied TNKS served as a potential miR-149-5p target. The direct interaction was further determined by miRNA pull-down assay (Fig. 5g, h), suggesting that TNKS, targeted by miR-149-5p, was an important downstream effector of circ5615.

Deregulated Wnt pathway activity implicates in various cancers and ninety percent of CRC patients have a mutation in key regulatory factors of the Wnt/β-catenin pathway, leading to activation of the pathway^44–47^. As TNKS is identified to stabilize β-catenin by stimulating AXIN degradation, multiple small-molecule TNKS inhibitors have been developed, however, there remain limits on clinical application^28,29,48,49^. Both western blot and Immunofluorescence assays demonstrated circ5615 had effects on Wnt/β-catenin pathway (Fig. 6a, d and Supplementary Fig. 4d). Furthermore, rescue experiment revealed that circ5615 significantly attenuated the suppression of miR-149-5p on TNKS and knockdown of *TNKS* significantly attenuated the effects of circ5615 on Wnt/β-catenin signaling (Fig. 6g), suggesting that circ5615 promoted CRC progression via a miR-149-5p-TNKS-dependent mechanism. Crucially, application of therapeutic ASO targeting circ5615 significantly reduced tumor volume with the effect on Wnt signal pathway, providing new insight into TNKS-suppressive therapy.

In conclusion, our study demonstrated that circ5615 competitively bounded miR-149-5p to inhibit the suppressing effect on TNKS, then promoted CRC progression via Wnt/β-catenin pathway. Our findings expanded the understanding of the underlying mechanism of CRC and provided a novel potential target for CRC.

## Materials and methods

### Tissues and cell lines

All paired samples of tumorous tissues (T) and adjacent nontumorous tissues (N) were obtained from surgical resections of CRC patients without preoperative treatment at the Department of Thoracic Surgery, Nanjing Medical University Affiliated Cancer Hospital (Nanjing, China). The samples were confirmed by two experienced pathologists independently and stored at −80 °C in RNAlater Stabilization Solution (Invitrogen, USA) until use. Written informed consent was obtained from all patients. Collection of human tissue samples wad conducted in accordance with the International Ethical Guidelines for Biomedical Research Involving Human Subjects. This study was approved by the Ethics Committee of the Nanjing Medical University Affiliated Cancer Hospital and was performed in accordance with the provisions of the Ethics Committee of Nanjing Medical University.

Human CRC cells (HCT 116, LoVo, HT-29, and SW480) were provided by Stem Cell Bank, Chinese Academy of Sciences. NCM460 and HEK-293T were obtained from Jiangsu Key Lab of Cancer Biomarkers of Nanjing Medical University. HCT 116; HT-29 and NCM460 cells were cultured in McCoy’s 5A medium; LoVo cell was cultured in F-12K medium and HEK-293T was cultured in DMEM medium with 10% FBS. They were all cultured at 37 °C with 5% CO2. SW480 cell was cultured in Leibovitz’s L-15 medium with 10% FBS in a free gas exchange with atmospheric air. All cells were tested negative for mycoplasma contamination.

### CircRNA microarray analysis

For circRNA microarray analysis, total RNAs were isolated from the paired tissue samples of five CRC patients by TRIzol reagent (Invitrogen). The sample preparation and microarray hybridization were performed based on the Arraystar’s standard protocols. Briefly, total RNAs were digested with Rnase R (Epicentre, USA). Then, the enriched circular RNAs were amplified and transcribed into fluorescent cRNA. The labeled cRNAs were hybridized onto the Arraystar Human circRNA Array (8 × 15K, Arraystar). After having washed the slides, the arrays were scanned by the Agilent Scanner G2505C. Agilent Feature Extraction software was used to analyze acquired array images. Quantile normalization and subsequent data processing were performed using the R software package. Significant differential expressed transcripts were screened by fold change ≥1.5 or ≤0.67 and *p*-value ≤0.05.

For mRNA microarray analysis, total RNAs was amplified and transcribed into cRNA, which was then labeled and purification. After hybridized onto he Affymetrix PrimeView Human Gene Expression Array and washed, the arrays were scanned by GeneChip Scanner 3000 and analyzed with the Affymetrix GeneChip Command Console Software. Quantile normalization and subsequent data processing were performed using the SAM R software package.

### RNA sequencing analysis

Total RNA in SW480 cells with circ5615 knockdown was isolated using the Trizol (invitrogen) according to the manufacturer’s protocol. After assessment of RNA purity and integrity, rRNAs were removed from Total RNA (EpicentreRibo-Zero rRNA Removal Kit, illumine, USA). Subsequently, the purified RNAs were subjected to first strand and second strand cDNA synthesis following by adapter ligation and enrichment with a low-cycle according to instructions of NEBNext Ultra RNA Library Prep Kit for Illumina (NEB, USA). The purified l library products were evaluated using the Agilent 2200 TapeStation and Qubit2.0(Life Technologies, USA) and then diluted to 10 pM for cluster generation in situ on the pair-end flow cell followed by sequencing (2 × 150 bp) HiSeq3000. HISAT2 was used to align the clean reads to the mouse reference genome mm10 with default parameters. HTSeq was subsequently employed to convert aligned short reads into read counts for each gene model. Differential expression was assessed by DEseq using read counts as input. The Benjamini–Hochberg multiple test correction method was enabled. Differentially expressed genes were chosen according to the criteria of fold change >2 and adjusted *p*-value < 0.05.

### Preparation of RNA and RT-PCR

Total RNAs were extracted from cells/tissues using Trizol reagent (Invitrogen) according to the manufacturer’s instruction. The subcellular localization was detected using PARIS Kit (Ambion, USA). Genomic DNA (gDNA) was extracted from cells according to the PureLink Genomic DNA Mini Kit protocol (Invitrogen). For RNase R treatment, 2 μg of total RNA was incubated 30 min at 37 °C with or without 3 U/μg of RNase R (Epicentre), and the resulting RNA was subsequently purified using an RNeasy MinElute cleaning Kit (Qiagen, USA). RT-PCR were performed as described previously^16^. The cDNA and gDNA PCR products were investigated using 2% agarose gel electrophoresis. *GAPDH*, *ACTB*, and *snRNA U6* were used as internal standards. For calculation of circ5615 and miR-149-5p copy numbers, amplified circ5615 and miR-149-5p form cDNAs were purified, serially diluted to be as templates for RT-PCR. Standard curves were drawn according to the Ct values at different concentrations. Primers and oligonucleotide sequence are listed in Supplementary Table 4.

### Actinomycin D assay

HCT 116 cells were exposed to 2 μg/ml actinomycin D (Sigma, USA) at indicated time point. Then the cells were harvested, and total RNA was extracted. The stability of circ5615 and *NFATC3* mRNA was analyzed by RT-PCR.

### Oligonucleotide and vector transfection

siRNA, ASO, and miRNA mimics were synthesized by Ribobio (China). The cells were transfected using Lipofectamine RNAiMax (Invitrogen). For circ5615 expression vector, the full-length cDNA of human circ5615 was synthesized and cloned into the expression vector pAV-circRNA (Vigene, China). The final construct was verified by sequencing.

For luciferase reporter vector, the sequence of circ5615 and *TNKS* 3′UTR was cloned into the downstream of pGL3-promoter. Mutations of miRNA-binding sites in circ5615 and *TNKS* 3′UTR sequence were generated using Mutagenesis Kit (Vazyme, China). Cells were transfected using Lipofectamine 3000 (Invitrogen) and harvested for experiment after 24 h.

### Fluorescence in situ hybridization and Immunofluorescence

FAM-labeled circ5615 probe and CY3-labeled miR-149-5p probe were synthesized by Servicebio (China). Briefly, samples were fixed in 4% paraformaldehyde and digested with proteinase K. After prehybridization for 1 h, samples were hybridized with specific probes at 37 °C overnight. For Immunofluorescence, cells were fixed, washed three times with PBS and blocked in BSA. After incubated overnight at 4 °C with primary antibodies in blocking buffer, cells were washed three times with PBS and incubated with fluorophore-conjugated secondary antibodies in blocking buffer for 1 h at room temperature. Cell nuclei were stained with DAPI. After washed, the images were acquired by Zeiss LSM710 confocal microscope system (Leica Microsystems, USA).

### Cell proliferation, cell cycle, and apoptosis assays

Cell proliferation was examined using EdU assay (RiboBio) and real time cell analyzer system (ACEA Biosciences, USA) following the research protocol afforded by the manufacturer. Colony formation assays were performed to monitor CRC cell cloning capability. For cell cycle analysis, cells were labeled with PI/RNase Staining Buffer (BD Bioscience, USA) according to the manufacturer’s instructions. The DNA content was determined using flow cytometry (FACScan; BD Biosciences) equipped with CellQuest software (BD Biosciences). For cell apoptosis assay, cells were double stained with Annexin-V-PE (BD Biosciences) and propidium iodide (PI) (Sigma) following the manufacturer’s instructions. Finally, cell apoptosis was analyzed by FACS scan flow cytometer.

### Tissue microarray and chromogenic in situ hybridization

Tissue microarray (TMA) was performed as described previously^50^ and constructed by ninety-nine pairs of colorectal cancer tissues and adjacent nontumor tissues. For CISH, digoxigenin-labeled probe targeted circ5615 (5′-TCTGGCTCAAGATCGAAATATAGGGGTG-3′) was synthesized. Briefly, after dewaxing and rehydration, the samples were digested with proteinase K, fixed in 4% paraformaldehyde, prehybridized at 37 °C and hybridized with the digoxin-labeled probe overnight at 55 °C. The samples were washed and then incubated at 37 °C with an anti-digoxin mAb (Roche, USA). The sections were stained with Diaminobenzidine (DAB, DAKO, Denmark) and observed.

### RNA immunoprecipitation

The EZMagna RIP Kit (Millipore, USA) was used following the manufacturer’s protocol. Cell extract was incubated with magnetic beads conjugated with AGO2 or IgG antibody (Millipore). The beads were washed and incubated with Proteinase K to remove proteins. Finally, purified RNA was detected by RT-PCR analysis.

### RNA pull-down assay

Biotin-labeled circ5615 probe (5′-ATCTGGCTCAAGATCGAAAT-3′-biotin) was synthesized by RiboBio, and the assay was performed as previously described^51^. Briefly, SW480 cells were fixed by 1% formaldehyde for 10 minutes, lysed, and sonicated. After centrifugation, 20 μL of the supernatant was retained as input and the remaining part was incubared with a circ5615-specific probe-streptavidin M-280 dynabeads (Invitrogen) mixture overnight at 30 °C. The beads-RNA mixture was washed and incubated with 200 μl of lysis buffer and proteinase K to reverse the formaldehyde crosslinking. Finally, the mixture was added with Trizol for RNA extraction and detection. For miRNA pull-down assays, 3′ end biotinylated miRNA Mimic (RiboBio) was transfected into CRC cells at a final concentration of 20 nM by RNAiMax (Invitrogen) following the manufactures protocol. After 24 h, cells were lysed in lysis buffer described above and the same pull-down procedure was performed.

### Luciferase activity assays

HEK-293T cells were seeded in 96-well plates at a density of 5 × 10^3^ cells per well. After 24 h, the cells were co-transfected with a mixture of 50 ng Firefly luciferase reporter vectors, 5 ng Renila luciferase reporter vectors, and miRNA mimics at the indicated concentration. The luciferase activity was measured with Dual Luciferase Assay Kit (Promega, USA). For comparison, the Firefly luciferase activity was normalized relative to Renila luciferase activity.

### Western blot analysis

Western blotting were performed according to standard protocols as described previously^50^ and primary antibodies used were listed as follows: TNKS (proteintech, 18030-1-AP, China), AXIN2 (proteintech, 20540-1-AP), β-catenin (Cell Signaling Technology, 8480, USA), CCND1 (Abcam, ab134175, USA), Fibrillarin (Abcam, ab166630), GAPDH (Cell Signaling Technology, 2118S), and β-actin (Abcam, ab6276).

### Xenograft model

Animal experiments were conducted according to the Institute for Laboratory Animal Research Guide for the Care and Use of Laboratory Animals and followed protocols approved by the Animal Committee of Nanjing Origin Biosciences. In total, 4–6-week-old BALB/c male nude mice (Beijing Vital River Laboratory Animal Technology, China) were used for the xenograft model. In all, 5 × 10^6^ HCT 116 cells transfected with circ5615 overexpressed vector or control vector were suspended in 200 μl PBS and inoculated into the right flank of the mice. Mice were monitored twice every week for tumor growth. After 30 days the mice were sacrificed. For xenograft model of circ5615 ASO: 5 × 10^6^ SW480 cells were subcutaneously injected into a single flank of the mice. Two weeks later, mice with palpable tumors were randomly divided into two groups, 30 nmol circ5615 ASO-circ5615 and ASO-NC was intratumorally injected into the two groups twice per week for 3 weeks. After mice were killed, tumors were weighed and processed for further histological analysis. Tumor volume was calculated as follows: *V* (volume) = (length × width^2^)/2.

### Statistical analysis

Results are presented as mean ± standard deviation of the mean. Statistical analyses were performed using SPSS 25 software (Abbott Laboratories) and differences between groups were assessed by Student’s *t*-test. A probability of 0.05 or less was considered statistically significant.

## Supplementary information

Supplementary Table 1

Supplementary Table 2

Supplementary Table 3

Supplementary Fig. 1 Validation of circRNA candidates.

Supplementary Fig. 2 circ5615 promoted the malignant progression of CRC cells.

Supplementary Fig. 3 circ5615 functioned as a ceRNA binding to miR-149-5p.

Supplementary Fig. 4 circ5615 regulated TNKS expression activating Wnt/β-catenin pathway.

Supplementary information
