# Supplementary material for: circ5615 functions as a ceRNA to promote colorectal cancer progression by upregulating TNKS
Source: Cell Death Dis. 2020 May 11;11(5):356. doi: 10.1038/s41419-020-2514-0 (PMC7214456; doi:10.1038/s41419-020-2514-0)
Supplement: Supplementary file 8 — Supplementary information [file 41419_2020_2514_MOESM8_ESM.docx]

**Supplementary Fig. 1 Validation of circRNA candidates.**

**a** Volcano plot showed dysregulated circRNAs. T tumor tissue, N nontumorous tissue. **b** Detection of circRNAs and their corresponding linear mRNA after RNase R treatment by RT-PCR analysis. **c**, **d** Expression levels of hsa_circ_0000467 and hsa_circ_0045932 in 35 CRC samples and adjacent nontumor tissues were determined by RT-PCR. *ACTB* was used as a loading control. **e** The genomic loci of circ5615 and repeating elements (upper). BLAST alignment revealed the highly reverse complement of the IRAlus (lower). **f** Correlation analysis of circ5615 and NFATC3 mRNA in CRC tissues. **g** RT-PCR analysis revealed circ5615 expression was much higher than NFATC3 in CRC tissues. Data are shown as mean ± SD. *p < 0.05; **p < 0.01; ***p < 0.001, paired t-test.

**Supplementary Fig. 2 circ5615 promoted the malignant progression of CRC cells.**

**a** The interfering efficiencies of siRNA in HCT 116 cells. **b** Levels of circ5615 and NFATC3 in HCT 116 cells after circ5615 overexpression. **c** circ5615 facilitated the proliferation of HCT 116 cells shown by the RTCA assays with silencing or overexpressing circ5615. **d** Apoptosis analysis of SW480 cells with silencing or overexpressing circ5615. **e**, **f** circ5615 induced migration of SW480 (e) and HCT 116 (f) cells by the Matrigel assays. The cell numbers were determined using the ImageJ program. Data are shown as mean ± SD (n=3) or typical photographs of one representative experiment. Similar results were obtained in three independent experiments. *p < 0.05; **p < 0.01; ***p < 0.001; ns, nonsignificant, paired t-test.

**Supplementary Fig. 3 circ5615 functioned as a ceRNA binding to miR-149-5p.**

**a** Volcano plot showed dysregulated miRNAs using TCGA data. **b** Expression levels of miR-149-5p, miR-331-3p and miR-3944-3p in SW480 cells transfected with miRNA mimics. **c** RTCA proliferation analysis for SW480 cells transfected with miR-331-3p or miR-3944-3p. **d** Schematic model and sanger sequencing for wild type or mutant transcripts of circ5615 luciferase reporters. Data are shown as mean ± SD (n=3). *p < 0.05; **p < 0.01; ***p < 0.001, paired t-test.

**Supplementary Fig. 4 circ5615 regulated TNKS expression activating Wnt/β-catenin pathway.**

**a** The expression of genes after circ5615 knockdown or overexpression in HCT 116 cells. **b** RT-PCR analysis revealed TNKS, LUC7L3 and SHROOM2 expression were significantly decreased by miR-149-5p in HCT 116 cells. **c** Schematic model and sanger sequencing for wild type or mutant transcripts of TNKS 3’-UTR luciferase reporters. **d** The protein levels of TNKS, AXIN2, β-catenin and CCND1 in the CRC cells with overexpression of circ5615. Fibrillarin served as a specific nuclear marker. **e** The mRNA (upper panel) and protein levels (lower panel) of TNKS in the SW480 cells with knockdown of TNKS. **f**, **g** The mRNA (upper panel) and protein levels (lower panel) of TNKS (f) and RTCA cell proliferation analysis (g) in HCT 116 cells transfected with miR-149-5p mimics alone or co-transfected with circ5615. Data are shown as mean ± SD (n=3) or typical photographs of one representative experiment. *p < 0.05; **p < 0.01; ***p < 0.001; ns, nonsignificant, paired t-test.
